# Evaluating the Efficacy of Treatment with a GnRH Analogue in Patients with Central Precocious Puberty

**DOI:** 10.1155/2015/247386

**Published:** 2015-10-13

**Authors:** H. Nur Peltek Kendirci, Sebahat Yılmaz Ağladıoğlu, Veysel N. Baş, Aşan Önder, Semra Çetinkaya, Zehra Aycan

**Affiliations:** Pediatric Endocrinology, Dr. Sami Ulus Women Health, Children's Training and Research Hospital, 06080 Ankara, Turkey

## Abstract

*Objective.* GnRH analogues (GnRHa) are used in the treatment of central precocious puberty (CPP). The purpose of this study was to evaluate the efficacy of treatment with a GnRHa (leuprolide acetate) in patients with CPP.* Subjects and Methods.* A total of 62 female child patients who had been diagnosed with CPP, rapidly progressive precocious puberty (RP-PP), or advanced puberty (AP) and started on GnRHa treatment (leuprolide acetate, Lucrin depot, 3.75 mg once every 28 days) were included in the study. The efficacy of treatment was evaluated with anthropometric data obtained, progression of pubertal symptoms observed, as well as GnRHa tests, and, when necessary, intravenous GnRH tests carried out in physical examinations that were performed once every 3 months.* Results.* In the current study, treatment of early/advanced puberty at a dose of 3.75 mg once every 28 days resulted in the suppression of the HHG axis in 85.5% of the patients.* Conclusion.* The findings of this study revealed that a high starting dose of leuprolide acetate may not be necessary in every patient for the treatment of CPP. Starting at a dose of 3.75 mg once every 28 days and increasing it with regard to findings in follow-ups would be a better approach.

## 1. Introduction

Puberty is the period when pulsatile release of the gonadotropin releasing hormone (GnRH) starts as a result of activation of Hypothalamic-Pituitary-Gonadal (HPG) axis and the secondary sex characteristics develop. Central precocious puberty (CPP) is defined as development of secondary sex characteristics in relation to activation of the HPG axis before the age of 8 years in girls and before the age of 9 years in boys [1]. GnRH analogues (GnRHa) represent the treatment of choice in central precocious puberty, because arresting pubertal development and reducing either growth velocity or bone maturation should improve adult height [2]. In order to reach the treatment goal, it is important to determine the ideal treatment dose for patients diagnosed with central precocious puberty and started on treatment with GnRHa. Insufficient treatment dose results in the progression of bone age and pubertal symptoms due to inadequate suppression of gonadotropins. On the other hand, high treatment doses lead to higher treatment costs, unnecessary exposure of patients to high drug doses, inhibition of growth by suppression of endogenous growth hormone secretion, and bone density to be lower than expected during puberty [3–7]. Reliable outcomes have been reported with the depot form of leuprolide acetate administered at a dose of 3.75 mg once every 28 days [8]. However, there is variation among clinics about the starting dose of leuprolide acetate. Lower doses (3.75 mg, 80–120 mcg/kg/28 days) are being preferred in European countries, while higher doses (7.5 mg, 200–300 mcg/kg/28 days) are being administered in the United States of America [3–7]. Taking into account these variations in GnRHa doses, the aim of the current study was to evaluate the efficacy of leuprolide acetate treatment at a dose of 3.75 mg once every 28 days.

## 2. Subjects and Methods

Female child patients who were admitted to Dr. Sami Ulus Women Health, Children's Training and Research Hospital Clinics of Pediatric Endocrinology with symptoms of early puberty and who were then diagnosed with central precocious puberty (CPP), rapidly progressive precocious puberty (RP-PP), or advanced puberty (AP) according to the criteria below and those who started GnRHa treatment were included in the study:

- Secondary sex characters developed before the age of 8 years (CPP).
- AP was diagnosed according to the appearance of breast buds between the ages of 8 and 10 years, accompanied by the presence of pubic or axillary hair and/or accelerated growth rate or bone age greater than 2 SD above chronological age [9].
- The diagnosis of RP-PP was based on the appearance of breast buds before 8 years of age accompanied by the presence of one or more of the following findings: menses, pubic hair, accelerated growth velocity, or bone age greater than 2 SD above chronological age [10].
- It is a basal LH level of ≥0,3 mIU/mL in patients diagnosed with precocious puberty (PP) or a peak LH response of ≥5 mIU/mL in standard intravenous GnRH tests.

Ethical approval for the study was obtained from Ankara University Medical Faculty Ethics Committee. Informed consent forms were obtained from the parents of patients who agreed to join the study after providing verbal information regarding the subject and aim of the study.

**Figure 1:**
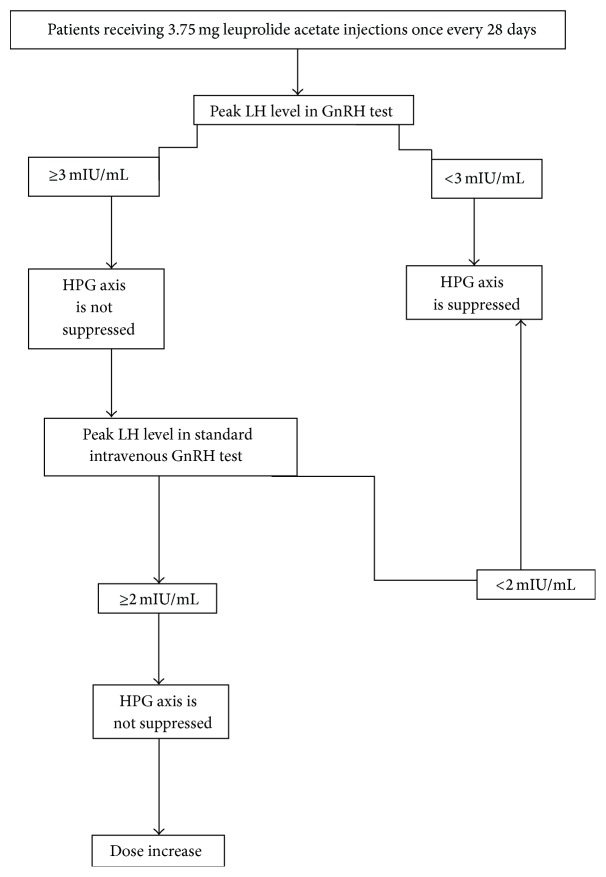
The flowchart used in the monitoring of the suppression of HPG axis and dose adjustments.

The study included 75 girls who are diagnosed as CPP/RP-PP or AP and started GnRHa treatment (leuprolide acetate, Lucrin depot, and intramuscular or subcutaneous injection 3.75 mg every 28 days). Written informed consent was obtained from parents. Thirteen patients were excluded because of less than 6 months of GnRHa therapy, irregular treatment and follow-up or incomplete anthropometric measurements, physical exam, or laboratory findings before or during therapy. Sixty-two girls who used GnRH analogue regularly for at least 6 months, followed regularly, and had complete anthropometric measurements, physical exam, laboratory findings, and left wrist X-rays before and during therapy were included. Detailed medical histories of all cases were recorded at the time of application. Physical examinations were made prior to and during the course of treatment at each follow-up visit once every 3 months, and staging of puberty was determined according to the criteria by Marshall and Tanner [11]. The chronological ages, nature and duration of complaints, and personal and family histories of patients were recorded at the time of application. In order to evaluate the efficacy of the treatment, suppression of HPG axis was monitored with GnRHa tests at intervals of 3 months. Blood samples were collected for the measurements of basal LH, FSH, and E_2_ levels prior to intramuscular leuprolide acetate injections and serum LH levels at 30 and 60 minutes following the injections. A peak LH response of <3 mIU/mL was accepted as the diagnostic criteria for suppressed HPG axis [12]. Patients with peak LH levels of ≥3 mIU/mL were suspected to have nonsuppressed HPG axis, and, thus, the HPG axis of these patients was reassessed with standard intravenous GnRH tests 3 weeks after the GnRHa injection. The standard GnRH test included taking blood samples for the measurement of basal FSH, LH, and E_2_ levels and intravenously (IV) administering 100 *μ*g of GnRH (gonadorelin acetate, Ferring Standard). Blood samples were retaken at 15, 30, 45, 60, and 90 minutes following the injection for the measurement of LH and FSH levels. The criterion for a suppressed HPG axis was a peak LH level of <2 mIU/mL in this test [13–15], and the leuprolide acetate doses for patients with a peak LH level of ≥2 mIU/mL were increased up to 7.5 mg every 28 days. The flowchart used in the monitoring of the suppression of HPG axis and dose adjustments is presented in Figure 1.

LH, FSH, and E_2_ levels were measured using the immunochemiluminometric (ICMA) assay with an Advia Centaur immunoanalyzer. The SPSS 16.0 statistics package program was used in the evaluation of the data; arithmetic means, standard deviation (SD), minimum-maximum limits, and significance levels (*p* values) were determined. Values are presented as mean ± SD or median and range (min-max). A significance level of 0.05 (*p* < 0.05) was chosen for all statistical analyses. Paired-*t* test was used if the distribution was normal in the evaluation of variation between the means of anthropometric and laboratory measures before and after GnRHa treatment, and Wilcoxon Test was used if the distribution was abnormal.

## 3. Results

The total of 62 female patients with CPP/RP-PP or AP had a mean age of 7.9 ± 1.3 (4.3–10.0) years and a mean bone age of 9.5 ± 1.9 (4.1–12.0) years at the beginning. Patients are diagnosed as 33.8% (*n* = 21) CPP, 33.8% (*n* = 21) RP-PP, and 32.4% (*n* = 20) AP. Initial clinical and hormonal characteristics of the patients are shown in Table 1. The decreases in basal and peak LH, basal FSH, and E_2_ levels on treatment were statistically significant (Table 2).

The dose of 3.75 mg once every 28 days resulted in the suppression of HPG axis in 53 patients (85.5%). The dose was increased to 7.5 mg starting in the 3rd month of treatment in 9 patients (14.5%) with nonsuppressed HPG axis. In the 6th month of treatment, the IV GnRH tests revealed HPG axis suppression in 5 of the 9 patients (8.0%), and because puberty halted or regressed, rate of growth slowed down, and there was no rapid progression of bone age, as observed in anthropometric findings and physical examinations, these patients were only followed up without an increase in drug dose. In the 9th month of treatment, suppression of HPG axis was not achieved in 3 patients (5.7%), but because there was no clinical progression of puberty, the drug dose administered to these patients was left unchanged. It was observed that the number and ratio of patients with nonsuppressed HPG axis decreased in inverse proportion to the duration of the treatment and that, at the end of the 12th month of treatment and afterwards, HPG suppression was achieved in all patients. The start of treatment and follow-up (while continuing GnRH treatment) characteristics of patients with nonsuppressed HPG axis during the course of treatment are presented in Table 3, and the distribution of these patients according to subgroups is shown in Table 4.

The characteristics of patients with and without HPG axis suppression (Table 5) revealed significantly higher pretreatment basal LH and E_2_ levels in patients without HPG axis suppression. In addition, the drug dose was very low in these patients, who were older and weighted more than the others.

## 4. Discussion

Leuprolide acetate treatment with a dose of 3.75 mg once every 28 days was initially started in all patients and, utilizing the dose-titration method, the dose was increased in patients who did not present sufficient suppression of HPG axis. This treatment regime resulted in suppression of the HPG axis in 85.5% of the patients, while an increase in dose was necessary in the remaining 14.5 percent. It was determined that the pretreatment basal LH and E_2_ levels of patients who did not present suppression of the HPG axis were significantly high because of the fact that most of these patients had advanced puberty. In addition, the drug dose was very low in these patients, who were older and weighted more than the others. Although there are various approaches to the GnRH dose used in the treatment of patients with CPP, the leuprolide acetate dose of 3.75 mg once every 28 days has been reported to adequately suppress LH and FSH levels [6, 16]. Higher treatment doses are preferred in the United States of America, while lower doses are used in Europe [8, 17–19]. The starting dose is either 300 mcg/kg/day or 7.5 mg minimum and 15 mg maximum every 28 days in the US in general, while a starting leuprolide acetate dose of 3.75 mg administered intramuscularly or subcutaneously is widely accepted in Europe [6, 8, 18, 19]. Similar to the findings of the current study, in a study by Carel et al., prepubertal responses were observed in the GnRH tests in the 3rd month of treatment in 85% of patients, who were diagnosed with CPP and treated with a leuprolide dose of 3.75 mg administered once every 28 days, and the authors concluded that a low-dose treatment was effective in the majority of patients [6]. In a different study by Brito et al., the same dose of 3.75 mg every 28 days did not achieve pubertal suppression in 4% of the patients, and, in turn, the dose was increased to 7.5 mg every 28 days [5]. In line with the literature, the findings of the current study revealed that the drug dose sufficient enough to suppress the HPG axis can vary among individuals and that a leuprolide acetate dose of 3.75 mg every 28 days is effective in suppressing the HPG axis and halting the progression of pubertal symptoms in most patients.

There are various approaches to evaluating the efficacy of GnRH treatment of patients with CPP. The gold standard for evaluating patients' responses to treatment is to determine the LH levels through GnRH stimulation and use a cutoff value of 2 mIU/mL [13–15]. However, difficulties in obtaining synthetic GnRH, additional costs, time and work force spent for the test, and test itself being painful and troublesome have led to research of different methods for evaluating the HPG axis in patients undergoing GnRH treatment. It has been reported that the free leuprolide in the depot form of leuprolide acetate stimulates gonadotropins and that the suppression of gonadotropins can be evaluated by measuring the LH levels between the 30th and 120th minutes following injection. The LH level threshold for the suppression of gonadotropins following a leuprolide acetate injection has been suggested as 3 mIU/mL [12]. However, a study in which the GnRH test results were compared with the night LH profiles of the same patients revealed that even though pubertal suppression was not observed in the GnRH test in these patients, prepubertal levels were determined in the night LH profiles in 33% of them. Therefore, it was recommended that patients that do not present HPG suppression when evaluating by measuring LH levels following GnRHa injection should be reevaluated with an IV GnRH stimulation test [20]. In the current study, when evaluating the efficacy of treatment, the patients were administered a GnRHa test with an interval of 3 months, a peak LH value of ≥3 mIU/mL was regarded as a nonsuppressed HPG axis, and patients with nonsuppressed HPG axis were administered an IV GnRH test in order to monitor pubertal suppression. Pubertal symptoms, anthropometric measurements, and bone age were also included in the assessments of the patients. It has been recommended that patients, who were diagnosed with CPP and were receiving GnRHa treatment, should be monitored by clinical evaluations of linear growth, sexual maturation, and bone age and that a halt or regression in the development of secondary sex characteristics, decrease in high growth rate, and deceleration of rapid progression of bone age should be regarded as signs of clinical response to the treatment [12, 21]. Therefore, in the current study, patients who did not present suppression of HPG axis in IV GnRH tests administered in the 6th and 9th months were followed up without an increase in treatment dose since they showed halted or regressed pubertal symptoms, decelerated rapid progression of bone growth, and growth rates reduced to prepubertal levels. Moreover, these patients were observed to have suppressed HPG axis at the end of 12 months of treatment.

The findings of the current study revealed that a high starting dose of leuprolide acetate in patients with CPP may not be necessary and that starting the treatment with a dose of 3.75 mg every 28 days and then increasing the dose during the follow-up period, if necessary, would be a more favorable approach. Additionally, it should be considered that higher doses may be required especially in the patients with advanced puberty. It is believed to yield better results when doses are calculated considering body weight in patients who have significantly high serum levels of LH and E_2_ and are older and therefore weighted more.

## Figures and Tables

**Table 1:** Initial clinical/hormonal characteristics of the patients and treatment dose (mean ± SD) (range).

	CPP (*n* = 21)	RP-PP (*n* = 21)	AP (*n* = 20)
Chronological age (years)	6.7 ± 1.1 (4.3–8.5)	7.9 ± 1.1 (4.5–9.4)	9.3 ± 0.5 (8.5–10.1)
Bone age (years)	7.7 ± 1.4 (4.1–10.0)	9.7 ± 1.4 (6.8–11.1)	11.2 ± 1.2 (8.8–12.0)
BMI SDS	0.5 ± 1.0 (−1.3–2.8)	1.0 ± 0.8 (−0.9–2.6)	0.9 ± 0.8 (−0.3–3.3)
Stage of puberty (median) (range)	2 (2-3)	3 (2-3)	3 (2–5)
Basal LH (mIU/mL)	0.2 ± 0.3 (0.1–1.5)	0.8 ± 0.9 (0.1–2.9)	2.6 ± 2.3 (0.1–10.3)
Peak LH (mIU/mL)	8.0 ± 5.1 (5.3–27.1)	9.8 ± 11.1 (5.0–48.8)	17.6 ± 12.4 (9.8–32.0)
E_2_ (pg/mL)	20.4 ± 11.7 (3.8–54.9)	25.3 ± 11.7 (17–51.3)	37.7 ± 15.9 (20.0–68.7)
Leuprolide acetate dose (mg/kg/28 days)	0.14 ± 0.03 (0.11–0.23)	0.12 ± 0.02 (0.09–0.19)	0.09 ± 0.01 (0.04–0.13)

CPP: central precocious puberty, RP-PP: rapidly progressive precocious puberty, AP: advanced puberty, BMI: body mass index, and SDS: standard deviation score.

**Table 2:** Basal and stimulated LH, FSH, and E_2_ levels of patients and leuprolide acetate dose at the start and during the course of treatment (mean ± SD) (range).

Hormones	Initially (*n* = 62)	After treatment with GnRH analogue							
		3rd month (*n* = 62)	*p* ^*∗*^	6th month (*n* = 62)	*p* ^*∗*^	9th month (*n* = 52)	*p* ^*∗*^	12th month (*n* = 34)	*p* ^*∗*^
Basal LH (mIU/mL)	1.2 ± 1.7 (0.07–10.3)	0.2 ± 0.2 (0.07–1.1)	0.00	0.2 ± 0.1 (0.07–0.6)	0.00	0.2 ± 0.1 (0.07–0.6)	0.00	0.2 ± 0.1 (0.07–0.9)	0.00
Peak LH (mIU/mL)	9.2 ± 9.0 (3.8–48.8)	2.0 ± 1.4 (0.4–8.4)	0.00	1.7 ± 1.1 (0.3–6.0)	0.00	1.6 ± 0.7 (0.1–4.2)	0.00	1.5 ± 0.6 (0.4–2.9)	0.00
Basal FSH (mIU/mL)	3.7 ± 2.1 (0.7–8.9)	2.2 ± 1.1 (0.4–4.3)	0.00	2.3 ± 1.0 (0.4–3.7)	0.00	2.3 ± 0.9 (0.2–3.3)	0.00	2.7 ± 0.7 (1.1–5.1)	0.15
E_2_ (pg/mL)	27.6 ± 14.9 (3.8–68.7)	17.0 ± 6.9 (7.0–32.6)	0.00	17.0 ± 7.4 (7.0–36.9)	0.00	15.6 ± 7.1 (7.0–33.7)	0.00	14.2 ± 7.4 (7.0–39.8)	0.00
Leuprolide acetate dose (mg/kg/28 days)	0.12 ± 0.03 (0.04–0.23)	0.13 ± 0.05 (0.04–0.33)	0.03	0.12 ± 0.04 (0.04–0.32)	0.00	0.12 ± 0.05 (0.04–0.31)	0.00	0.12 ± 0.04 (0.04–0.32)	0.00

The *p* ^*∗*^ value represents the difference according to value before treatment.

**Table 3:** Characteristics of patients without HPG axis suppression (mean ± SD) (range).

Treatment duration	Chronological age (years)	BMI SDS	Stage of puberty (median) (range)	Basal LH (mIU/mL)	Peak LH (mIU/mL)	E_2_ (pg/mL)
Pretreatment (*n* = 9)	8.6 ± 1.0 (7.0–10.1)	0.9 ± 0.8 (0.7–2.2)	3 (2–4)	2.2 ± 3.3 (0.1–10.3)	11.1 ± 10.6 (5.6–27.1)	32.9 ± 24.0 (7.0–68.3)
3rd month (*n* = 9)	8.9 ± 1.0 (7.3–10.4)	0.8 ± 0.7 (0.7–2.0)	3 (1–4)	0.1 ± 0.0 (0.1–0.2)	4.7 ± 1.9 (3.0–8.4)	13.7 ± 6.9 (7.0–22.6)
6th month (*n* = 5)	9.5 ± 1.2 (7.5–10.5)	0.9 ± 0.6 (0.8–2.1)	3 (2–4)	0.4 ± 0.1 (0.2–0.6)	4.8 ± 0.8 (3.8–6.0)	14.1 ± 9.8 (7.0–30.8)
9th month (*n* = 3)	9.7 ± 1.6 (7.8–10.8)	0.9 ± 1.0 (0.8–2.2)	3 (2–4)	0.4 ± 0.1 (0.4–0.6)	3.8 ± 0.3 (3.6–4.2)	18.6 ± 7.6 (11.8–26.8)

BMI: body mass index; SDS: standard deviation score.

**Table 4:** The distribution of the patients without HPG axis suppression according to subgroups (*n*) (%).

Treatment duration	CPP (*n* = 21)	RP-PP (*n* = 21)	AP (*n* = 20)
3rd month	2 (9.5%)	2 (9.5%)	5 (25%)
6th month	1 (5%)	0	4 (20%)
9th month	0	0	3 (15%)
12th month	0	0	0

CPP: central precocious puberty, RP-PP: rapidly progressive precocious puberty, and AP: advanced puberty.

**Table 5:** Characteristics of patients with and without HPG axis suppression (mean ± SD) (range).

Characteristic	HPG axis		*p*
	Suppressed (*n* = 53)	Nonsuppressed (*n* = 9)	
Chronological age (years)	7.8 ± 1.4 (4.3–9.9)	8.6 ± 1.0 (7.0–10.1)	0.32
Bone age (years)	9.3 ± 2.0 (4.1–11.1)	10.6 ± 1.4 (7.8–12.0)	0.16
BMI SDS	0.8 ± 0.9 (−1.4–1.6)	0.9 ± 0.8 (−1.0–3.3)	0.95
Basal LH (mIU/mL)	1.0 ± 1.2 (0.07–4.6)	2.2 ± 3.3 (0.1–10.4)	0.005
Peak LH (mIU/mL)	9.0 ± 8.9 (3.9–48.8)	11.1 ± 10.6 (5.6–27.1)	0.44
E_2_ (pg/mL)	26.7 ± 12.9 (3.8–58.5)	32.9 ± 24.0 (7.0–68.7)	0.00
Leuprolide acetate dose (mg/kg/28 days)	0.13 ± 0.14 (0.04–1.12)	0.11 ± 0.02 (0.09–0.17)	0.47

BMI: body mass index; SDS: standard deviation score.
